# Transgender women in Malaysia, in the context of HIV and Islam: a qualitative study of stakeholders’ perceptions

**DOI:** 10.1186/s12914-017-0138-y

**Published:** 2017-10-18

**Authors:** Sima Barmania, Syed Mohamed Aljunid

**Affiliations:** 1 UKM Medical centre, Faculty of Medicine, International Centre for Casemix and Clinical Coding, National University of Malaysia, Jalaan Yaacob Latif, Kuala Lumpur, 56000 Cheras Malaysia; 2 United Nations University International Institute for Global Health, Kuala Lumpur, Malaysia; 3 Department of Health Policy and Management, Faculty of Public Health, Kuwait University, Kuwait City, Kuwait; 4 Institute of Education, University College London, London, United Kingdom

## Abstract

**Background:**

Globally, one of the key groups considered to be at high risk of acquiring HIV are transgender women, often a marginalised group. In the Malaysian context there has been a scarcity of published research relating to transgender women, a sensitive issue in a Muslim majority country, where Islam plays an influential role in society. Furthermore, there has been a paucity of research relating to how such issues relate to HIV prevention in transgender women in Malaysia.

Thus, the aim of this study is to explore the attitudes of stakeholders involved in HIV prevention policy in Malaysia towards transgender women, given the Islamic context.

**Methods:**

In-depth interviews were undertaken with stakeholders involved in HIV prevention, Ministry of Health, Religious Leaders and People Living with HIV, including transgender women. Thirty five participants were recruited using purposive sampling from June to December 2013 within Kuala Lumpur and surrounding vicinities. Interviews were in person, audiotaped, transcribed verbatim and used a framework analysis.

**Results:**

Five central themes emerged from the qualitative data; Perceptions of Transgender women and their place in Society; Reaching out to Transgender Women; Islamic doctrine; ‘Cure’, ‘Correction’ and finally, Stigma and Discrimination.

Discussion: Islamic rulings about transgenderism were often the justification given by participants chastising transgender women, whilst there were also more progressive attitudes and room for debate. Pervasive negative attitudes and stigma and discrimination created a climate where transgender women often felt more comfortable with non-governmental organisations.

**Conclusion:**

The situation of transgender women in Malaysia and HIV prevention is a highly sensitive and challenging environment for all stakeholders, given the Muslim context and current legal system. Despite this apparent impasse, there are practically achievable areas that can be improved upon to optimise HIV prevention services and the environment for transgender women in Malaysia.

## Background

Globally, one of the key groups considered to be at high risk of acquiring HIV are transgender women, defined for the purpose of this study, as women assigned the male gender at birth but who identify themselves as being women. Transgender women often represent a marginalised population and a ‘very high burden population for HIV’ [1]. In a comprehensive meta-analysis and systematic review conducted by Baral and colleagues, an assessment of the relative HIV burden in transgender women was undertaken including 15 countries which revealed a pooled HIV prevalence of 19.1% in 11,066 women worldwide and the odds ratio of being infected with HIV for transgender women compared with all adults of a reproductive age was 48.8 [1]. The authors deduce that there is an ‘urgent need of prevention’ in transgender women in a group that are often not included in national HIV surveillance and identify factors, such as engagement in high risk anal sex with men increasing their vulnerability, as well as stigma and discrimination in health care settings as hindrances to accessing HIV prevention services [1]. In the UNAIDS Prevention Gap Report, key populations such as transgender women are not reached by current prevention efforts [2]. The Asia Pacific Trans Blueprint for Health in Action clearly mentions ‘trans women as disproportionately affected by HIV—yet there are still insufficient programmes or services targeted to meet trans-specific need’ [3]. Studies have concluded that transgender women are at increased risk of HIV due to factors, such as high-risk sexual behaviour, commercial sex work and stigma and discrimination [4]. Operario and colleagues conducted a meta-analysis and found the prevalence of HIV was higher in transgender female sex workers compared to transgender women who did not engage in sex work, 27.3% and 14.7% respectively [5].

Furthermore, Transgender women often are at risk or have a high burden of health issues other than HIV such as mental health issues and substance misuse [6], as well as pervasive societal discrimination which can create a barrier to accessing health care [7]. This is the case globally as well as in the Asia-Pacific region in countries such as, Malaysia [8].

### Transgender women in Malaysia

In the Malaysian context, there has been a scarcity of published research relating to HIV amongst transgender women, a sensitive issue in the Muslim majority country [9]. However, it has been estimated that the prevalence of HIV amongst transgender women in Malaysia is 5.7% and it has been suggested that this could be underreported and underestimated [10], with newer reports suggesting dramatically higher prevalences in urban areas such as Kuala Lumpur [2].

The local term for male to female transgender women in Malaysia is ‘*Mak Nyah’* and earlier estimations suggested that there were between 10,000–20,000 in the country with the majority being Malay Muslims [11]. In 2002, Teh undertook the first large-scale study to assess HIV/AIDS knowledge amongst transgender women, studying 507 ‘*Mak Nyah’*, finding over 92% received payment for sex, although only 54% claimed they were sex workers [12]. Subsequent research involving 15 *‘Mak Nyah’* found that all respondents were aware of HIV/AIDS but lacked in depth information about the disease and did not regard ‘HIV/AIDS as a primary concern for them’, as their main immediate worries centred around financial problems, discrimination and ‘coping as a Mak Nyah in Muslim society’ [11]. In addition, although condoms were carried they were seldom used due to issues such as clients’ refusal, getting paid more for not using condoms, oral sex or perceptions of clients’ health [11].

In addition, within the last few years, more attention has been cast over the current treatment of the transgender community in Malaysia. Lee argues that historically transgender women were more culturally accepted in Malaysian society, even up until the 1980s [13]. However, recently widespread conservative Islam both socially and legally has resulted in a culture of ‘moral policing’ citing the example of a transgender woman who was assaulted by Islamic enforcement officers, sent to hospital due to her injuries and the staff told to write down the name of any transgender people who visited her [13].

In Malaysia, Islam plays a strong role in influencing individual behaviour [14] as well as society, gender and sexuality [15]. Islamic rulings on transgender women and sex work are embedded in the legal structure, a dual system including criminal and Shariah law (Islamic law pertaining to Muslims only) which affects the law itself and regulation of that law by the police and relevant authorities.

The Syariah (Malaysian spelling of Shariah) Criminal Offences Federal Territories Act specifically mentions transgender women under Section 28 whereby “any male person who, in any public place, wears a woman’s attire and poses as a woman for immoral purposes shall be guilty of an offence and shall on conviction be liable to a fine not exceeding one thousand ringgit or to imprisonment for a term not exceeding one year or both” [16]. Section 29 of the Act refers to “any person who, contrary to Islamic Law, acts or behaves in an indecent manner in any public place” is guilty of an offence. The Act also refers to sex work which some transgender women may be engaged in “any woman who prostitutes herself shall be guilty of an offence” [16].

Added to this is the fact that in 1982 the decision was made by the National Fatwa Council prohibiting sex reassignment surgery under Islamic law, apart from intersex cases.

Research conducted amongst the Malaysian lesbian, gay, bisexual and transgender community, including 13 transgender women, found that participants were separately subjected to discrimination by Islamic religious officials and also by health providers with some participants feeling reluctant to access medical services due to either being verbally abused or ill-treated by health professionals [17]. It is worth pointing out that the aforementioned research noted that these health professionals were often Muslim and influenced by their own perception of transgender women, influenced by religious views as well as cultural and politics shifts, which can change over time. Furthermore, the cultural, and political climate influences how the religious community interpret opinions about transgender women in the context of Islam. In the Quran there is no explicit mention of transgender women, however various academics have commented on references in the Hadith, a secondary source of texts which report the actions and words of the Prophet Muhammad. One prominent academic includes Rowson, who describes the ‘existence of a form of publicly recognized and institutionalized effeminacy’ among men in pre and early Islamic society. [18]. These people were referred to as *mukhannathun* ‘effeminate ones’ and allowed to ‘associate freely with women, on the assumption that they had no sexual interest in them’ [18]. Also important says Altinay is the ‘different motivations to transgender expression, ‘innate gender identity’ or for the purposes of transgressing Islamic rulings with regards to conduct [19]. Furthermore, Alipour defines up to five manifestations of ‘gender ambiguity’ in premodern Muslim society (including mukhannathun) with these types not necessarily correlating with the definition of transgender women in the Western world [20].

Additionally, in 2014 the international non-governmental organisation (NGO) Human Rights Watch published a report recounting various allegations of human rights abuses experienced by transgender people in Malaysia [21]. Some of those issues catalogued directly related to healthcare, included refusing to touch transgender women as patients, as well as highlighting that many transgender women felt discriminated at school and had lower levels of education. More specifically, Human Rights Watch recommendations to the Ministry of Health included conduction of non-discrimination training for health personnel towards transgender people as well as off- site HIV testing in ‘safe spaces’ [21].

More recently, a qualitative study consisting of 21 in-depth interviews with Malaysian transgender women who were sex workers was undertaken which highlighted the stigma and discrimination faced when accessing appropriate health care [22]. The authors also conclude that while healthcare is accessible, the Malaysian government fails to provide the funding for HIV prevention which is specific for Mak Nyah and that the “healthcare and prevention system is poorly equipped to provide targeted HIV prevention and treatment” [22].

While the study adds to a steadily growing body of research relating to the individual and collective experiences of transgender women in Malaysia, there is a dearth of knowledge relating to stakeholders involved in HIV prevention policy and their attitudes to transgender women.

Thus, the aim of this study is to explore the attitudes of stakeholders involved in HIV prevention policy in Malaysia towards transgender women, given the Islamic context.

## Methods

This study is part of a broader research project looking at the role of Islam in shaping HIV prevention policy in Malaysia, a brief overview of which has been described previously [23]. The original study sought to understand how perceptions of Islam actually affect HIV prevention in Malaysia from a neutral, public health perspective, sensitive to the Islamic and political context by undertaking 35 semi structured interviews with the 3 key stakeholders identified as being involved in HIV prevention policy identified following a thorough review of the literature [23]. These included officials from the Ministry of Health staff, Religious leaders and People Living with HIV (PLHIV) including transgender women. This qualitative study looks solely at the sensitive issue of transgender women in Malaysia in the context of HIV and Islam as this was one of the central themes that emerged from the original study.

Participants were recruited using purposive sampling techniques, in-depth semi structured interviews with key stakeholders. Participants were recruited purposively from the vicinity of Kuala Lumpur and Putrajaya (Klang Valley) in Malaysia, from June to December 2013, with interviews conducted from October onwards after ethical approval was granted from UKM. This study was approved by the National University of Malaysia (UKM) UKMMC Research and Ethics committee and participants provided verbal and written informed consent. In total 35 participants were recruited, 19 representing PLHIV, 11 religious leaders and 5 officials from the Ministry of Health, including their personal opinion. People living with HIV were recruited through the network and assistance of local non- governmental organisation PT Foundation (formally known as Pink Triangle Foundation) with participants including men who have sex with men, sex workers, heterosexual women as well as 4 transgender women. Islamic religious leaders included those from the department of Religious Affairs (Jabatan Kemajuan Islam Malaysia (JAKIM), Shariah council, as well as Islamic academic scholars. Participants from the Ministry of Health included those at the national and state level, with saturation reached early. Inclusion criteria stipulated that participants resided in the Klang valley region were over the age of 18 and provided full informed consent. Criteria for exclusion for PLHIV participants were those who had been living with HIV for less than a year as well as those who most likely acquired HIV through intravenous drug use (as this constituted a different disease dynamic) selected by staff within the local non-governmental organisation. All interviews were conducted in person, face to face, by the same researcher (SB) using a topic guide which ensured standardisation. Informed consent was obtained in verbal and written form and participants were aware of the objectives of the study as well as confidentially. Interviews lasted between 60 to 90 min, were digitally recorded and were conducted in English, whilst on occasion, in-house translation was required. The interviewer made every effort to develop a rapport with participants and ensuring they felt comfortable and at ease; this included wearing religious appropriate attire (covering of the head and limbs) when meeting religious leaders. Non- verbal cues were documented during the interview as well as hand written notes and reflections made following the interview by the researcher. The digital audiotapes were transcribed by a professional transcriber, who was satisfied with both the quality and clarity of the recordings. Subsequently, a document of the transcribed verbatim was produced which was compared with the audio recordings to ensure the consistency of content. The process of analysis started relatively early and was conducted by the same researcher (SB) throughout, audio recordings were listened to and transcripts reread to familiarise the researcher with the data. Printed copies of the verbatim transcripts from 15 participants, comprising various stakeholders were annotated with themes and subthemes noted which created a framework analysis used to read and analyse all 35 interview transcripts.10 themes arose from the parent study, including one theme specifically focussing on transgender women, described in this study with 5 further subthemes presented in the results below.

## Results

The data from the 35 qualitative interviews revealed five principal themes: Perception and place; reaching out; Islamic doctrine; ‘correction’ and stigma and discrimination.

### Perceptions of transgender women and their place in society

It was observed that perceptions of transgender women in Malay society had changed over time, historically being more accepting of this group.

“Traditionally transgender people have been well accepted in Malay society; you will find them everywhere- you go to weddings, all the ones that do the makeup, the hair and all the decorations. So that’s part of our culture; that really has been an old part of our culture. It’s only now that it’s really become a ‘no no’ ” (IV 23 PLHIV).

The perception of transgender women in Malaysian society conveyed by participants across stakeholder groups was that they were considered a marginalised and vulnerable part of society. Transgender participants living with HIV who were interviewed were well aware of how they themselves were perceived in mainstream Malaysian society, often associated with illicit and criminal activities such as sex work.

Transgender women were also associated with performing certain sexual practices such as, giving oral sex to men as well as being receivers of anal sex.

“I guess there is a misconception in our society about transgenders, they are promiscuous and they are basically the vehicle for transmission of sexually transmitted diseases”. (IV 8 Religious Leader)

Amongst other participants living with HIV interviewed, there was an appreciation and sympathy that the transgender community was a highly visible, marginalised minority of society linked to ill health and sexual activity.

“….they [Transgender women] are equated to being sex workers” (IV 16 PLHIV).

“At the same time, in Malaysia, these transgenders are almost 85 % engaged with sexual activities”. (IV 3 Ministry of Health staff)

While there was extensive condemnation surrounding those transgender women who undertake sex work there was little discussion or intrigue regarding the identity of their clients’.

“…Transgender they never question the clients. Who are the clients? many of their clients are also Malay”. (IV 16 PLHIV).

However, not all transgender women were sex workers, stated one participant adamantly.

“Actually I am not a prostitute; I am working. Sometimes I thinking to God: ‘it’s not fair- you have given me this [HIV]” (IV 5 PLHIV TG).

The majority of transgender participants revealed feelings of being judged, either by Malaysian society in general or by religious leaders, with one participant explaining:

“The most important thing is to not judge people, judge people from the covers.” (IV 5 PLHIV TG)

Transgender participants felt ostracised from society and experienced demonization, particularly amongst religious leaders, with some having strong reactions towards them.

“They have their religion and tradition and I feel shy (*Malu*) to go [to a religious leader] as a transgender” (IV 6 PLHIV TG).

### Reaching out to transgender women

Transgenderism, or changing one’s gender was simply not recognised by religious leaders across the spectrum; so often there was scepticism and suspicion by the transgender community when religious leaders did make a concerted effort to reach out to them. Often help or assistance was associated with specific conditions, such as dressing as a man rather than their preferred state as women.

This scenario is described by a transgender woman, whom after encountering accommodation difficulties sought help from a caseworker who facilitated her being placed in a shelter house, this later became her conduit for interacting with religious leaders, whom she had an unfavourable experience with.

“And then she [translated via a friend] started seeking help from the caseworker but she was put in the house and that’s where she started to encounter the religious people. ‘You just be a man’. So that’s why she cut her hair. Although she looks like this [pointng to short hair] her heart’s still a woman…In the house, there are certain rules and she is engaging with the religious department. She is not comfortable in herself”. (IV 4 PLHIV TG)

The account described above (translated by a transgender staff member) was of particular interest, as this individual physically resembled a man, differing in appearance compared to the other transgender women interviewed in the study. The only reason for her masculine attire was due to the requirement of the shelter home, that she was not permitted to dress as a woman, a prerequisite for residency. These ‘conditions’ increased tensions and sullied the relationship with the transgender community and religious leaders.

The Ministry of Health also reached out to the transgender community with a focus on disease control and prevention of HIV.

“But as far as our Ministry is concerned, we do reach out for them in their various groups in terms of disease control, knowing that they have a certain high-risk lifestyle. So from that point of view, we do that.” (IV 11 Ministry of Health staff)

However, the Ministry of Health were faced with a number of difficulties when working with the transgender women, including accessing them and acknowledged that having contacts within the community with whom they could co-operate with was vital.

“There is a lot of challenges, we can’t reach them…Having contacts is very important with the transgender communities” (IV 25 Ministry of Health staff).

“Yeah, the challenge is for us to approach them and for them to be convinced that we are here to help prevent the spread of HIV” (IV 31 Ministry of Health staff).

“You have to participate with them, to contribute and that’s what I’m saying, you want to tackle HIV/AIDS then you have to work as a team, from different disciplines, each discipline will do its part and its accountability” (IV 31 Ministry of Health staff).

Nevertheless, there was often cognisance amongst participants from the Ministry of Health that ultimately the Ministry could not recognise transgender as an official gender.

“That is an issue, because, in the Muslim community, they don’t recognise that [transgender]. So when I say don’t recognise them, to accept them as a legal entity… it’s quite difficult. Or human talking about their rights and all those kind of things”. (IV 11 Ministry of Health staff)

“The minority transgender group is fighting for legal reform…which is impossible for Malaysia, because we are a Muslim country; we are not a secular country. They must understand that…our law, our regulations are based on the Qur’an and Sunnah. That’s it; it’s very clear”. (IV 10 Ministry of Health staff)

Often underlying these outlooks, by both religious leaders and the Ministry of Health, was perceptions of transgenderism in Islamic doctrine, which was often a reference point.

### Perceptions of Islamic doctrine

Typically participants articulated that changing one’s gender from male to female was forbidden in Islam and was outside the bounds of appropriate behaviour.

“There is no acceptance from Islam” (IV 4 PLHIV TG)

“….presenting as a woman, it’s not allowed…He is a man and he presents himself as a woman, maybe from his dressing sign or his body language sign…it’s not allowed” (IV 12 Religious Leader)

The Islamic view cited by participants was that men should not impersonate the characteristics of women, in terms of dressing and mannerism. However, a further reason mentioned was that any change to the body, such as tattooing, or plastic surgery was not permissible, with the rationale that doing so was changing the body which was given to them by Allah the creator.

“….We are not permitted to change ourselves”. (IV 12 Religious Leader)

Nonetheless, there was an appreciation that there was the existence of people of indeterminate sex (Khunsa), a medical condition which naturally exists in the wider population, in both Muslim and non-Muslim societies now and throughout history.

“Khunsa, in Malaysia, we are very strict in the Muslims scholars, the muftis, the Muslim preachers are very strict in that sense.... they only allow certain people under the category of Khunsa; you know Khunsa? A Khunsa who....person born with two sex organs so…and he or she has to, to make up his mind based on both emotional and also factors of whatsoever”. (IV 24 Religious Leader)

Religious leaders who participated in the study were resolute in their belief that the gender categories were either, male, female or Khunsa; the latter group having to choose the gender which suited them best, physically and emotionally. However, participants deemed that very few people in Malaysia could be categorized as ‘Khunsa’, instead were transgender, the former accepted whilst the latter was not.

“Whether they are being a man, or being a woman. So this is what they call as Khunsa. They have in the Qur’an…This is the Khunsa....different with the transgender… We recognise Khunsa but we do not recognise transgender”. (IV 2 Religious Leader)

However, there was a minority of religious leaders (two out of the eleven) who took an alternative view to their peers and considered a more flexible compassionate approach to transgender women, especially in cases where significant suffering was caused.

“Say he is supposed to be a woman, being trapped in a man’s body, then he should be allowed to have a sexual change. And I believe Iran in that aspect is more progressive than any other countries, any other Muslim countries in the world, yeah”. (IV 8 Religious Leader)

The excerpt above was certainly an atypical viewpoint, but some religious leaders believed that the local custom and culture had to also be taken into consideration.

“Here fatwa [prohibiting gender reassignment surgery] has been issued but if you go to countries, Muslim countries like Iran, for example, they are very flexible on this, I don’t say Malaysia is wrong on that perspective, this has to go back to the culture, the local culture”. (IV 24 Religious Leader)

Notwithstanding, many participants took a completely differing view to the ones described above and held a strong conviction that male to female transgender women could return to the gender assigned to them at birth.

### ‘Cure’, ‘correction’ and ‘coming back’

Some religious leaders, namely JAKIM had developed their own specific programmes to facilitate transgender women who wanted to be ‘cured’

“We have our own programme. JAKIM have developed a programme that we call Muhayam. Muhayam means camping”. (IV 2 Religious Leader)

Religious leaders who were involved in the Muhayam camp explained how they endeavoured to reach out to the transgender population by befriending them and using positive language.

“Cure. Information and cure. Transgender, we go to them, we teach them this is not good. We don’t say haram (forbidden), we do not; but this is not good. Man, why do you become a woman?” (IV 2 Religious Leader)

“We are going to teach them, we go and see them. You see, our concept is to be friendly with them, like a friend. So then, they are very happy to join our programme. We give solution, option, solution.” (IV 2 Religious Leader)

Religious leaders also felt that transgender women could become male and could be encouraged “to come back through Islamic teaching”. (IV 34 Religious Leader).

Whilst other religious leaders felt that there was a need to simply keep the numbers of transgender people “under control”. (IV 33 Religious Leaders).

JAKIM were clear to mention that these voluntary programmes were not ‘conversion camps’ as had been labelled by the transgender community, non-governmental and human rights organisations who were against such measures. However, there was support for these camps unofficially, amongst some participants, such as the Ministry of Health.

“But somehow in Islam, this issue [being transgender] can be corrected. I am very supportive of the ways JAKIM are trying to do the Muhayam activities, to bring back these people to be corrected into men or whatsoever”. (IV 3 Ministry of Health staff).

Transgender participants recalled that amongst religious leaders there was pressure to ‘come back’ to their original birth gender, were greeted with condemnation and questions as to why they were dressed in a certain way, making them feel unaccepted and highly uncomfortable.

“Ustads (religious leaders) definitely they will advise me, they will condemn me… Haram they say. Definitely, they will say, so you should come back, they will ask me to come to be a male, I feel more comfortable here with PT. I feel safe”. (IV 5 PLHIV TG)

The word ‘safe’ is poignant and mentioned repeatedly in the above excerpt by this transgender participant, thus many within the transgender community felt more at ease with non-governmental organisations, such as PT foundation, where they did feel safe.

However, the word ‘safe’ was often reiterated by a number of transgender participants exposing their vulnerability to stigma and discrimination.

### Stigma and discrimination

This stigma and discrimination were felt and manifested in a number of ways, across sectors, with transgender participants citing unfavourable experiences with not only the religious leaders but also the police, described below with the assistance of a translator.

“As a transgender in Malaysia, she went through a lot of risky stuff like police…with police, religious department…with the religious department; normally they chase them. You have to pay fine, sometimes, as usual, they ask you to come back to the path…And police will chase them”. (IV 7 PLHIV TG)

Even more, mistreatment was verbalised by transgender participants such as beatings, being stripped or made to show parts of their body against their will.

“I think one time only, one of the officer, a police, they stopped me, they caught me. Then they brought me to the police station, they asked me to go once to the toilet and asked me to open my backside. Then they beat me, they beat my backside, really. The police are so bad”. (IV 5 PLHIV TG)

“…Very bad, police…I think yeah, definitely, they will do to other transgenders…Because they discriminate people like us…something like we are alien”. (IV 5 PLHIV TG)

There were also issues raised about stigma and discrimination towards transgender women when utilising health care and this constituted a significant barrier to accessing treatment.

“ I don’t know what to put; female or male, sometimes they still discriminate us…No Doctor ok, I don’t trust the nurse, doctor ‘yes’, but nurse ‘no’, some of them are really bad mouthed- bad mouth, bad service”. (IV PLHIV 5 TG)

One particular issue raised was that transgender women were classified as men on their identity cards and as a result were placed in male wards, making them highly uncomfortable, as well as feeling discriminated against by health professionals.

These issues all added extra internal conflict for transgender women who in themselves feel very much that they were wholly women, in terms of attitude and lifestyle, however living in a society which sees them as men. As a result transgender communities were often driven underground and less likely to access mainstream health services and felt more comfortable with non-governmental organisation, such as PT foundation.

Most often transgender women were recommended to such organisations by their peers, other transgender women, where they obtained information about HIV.

“From the transgender women outside because sometimes when I go out, they say ‘come, I want you to come, there is people like us we can share everything’ ” (IV 5 PLHIV TG).

One transgender lady, who formerly was engaged in sex work, explained that she only had information about HIV once she was diagnosed as being HIV positive and that knowledge of condom usage could have prevented her getting the disease:

“If she knew about the usage of condoms earlier, she wouldn’t be in this situation now” (IV 4 PLHIV TG).

However, the environment is complicated by anecdotal reports and local media stories where condoms are used as evidence of sex work rather than as a HIV prevention strategy.

“In Malaysia, if you are carrying a condom inside your bag, if you are a woman, they will think you are a sex worker…they will make use of this condom as proof…evidence to bring you to court…but actually, it’s the way to protect themselves being infected with HIV”. (IV 22 PLHIV)

While another transgender woman explained that they did not know what HIV was before their diagnosis, but that subsequently through workshops organised by some of the non governmental organisations, she now knew about prevention:

“Not only HIV and AIDS but also STI, sexually transmitted diseases like syphilis and now she [translated via a friend] has the confidence to go and visit friends who are HIV positive” (IV 6 PLHIV TG).

## Discussion

There were various perceptions of transgender women amongst the stakeholders interviewed, some participants viewed transgender women as part of Malaysian culture, occupying a place in industries such as entertainment, hair and make-up. Whilst other stakeholders, namely religious leaders, considered transgender women negatively, associated with illegal activities, such as sex work, which has its own additional stigma attributed to it. This served to create an environment where transgender women were considered marginalised and thus a group which was hard to reach in terms of accessing HIV prevention activities. The Ministry of Health acknowledged that there were challenges accessing the transgender community and recognised the importance of contacts and partnership. In their engagement, they often reframed the issues towards prevention of illness and disease and steered away from debates on human rights and legal recognition of transgender women. This stance was primarily due to the fact that Malaysia was considered to be a Muslim country with the laws and regulations based on the Qur’an and Sunnah. The Ministry of Health are willing to help and reach out to the transgender women but their efforts are limited due to contextual issues such as cultural, legal and religious obstacles.

Indeed, Islamic rulings about transgenderism were often the justification given by participants chastising transgender women, especially by religious leaders. The predominant view was that in Islamic teachings emulating the characteristics of the opposite gender you were born with was not allowed or changing one’s gender. Additionally, there was the belief that even changing one’s appearance was not permissible as it was seen as challenging God’s role as the Creator. However, there was an acknowledgement of medical cases where an individual is born with ambiguous genitalia or hermaphrodites, known as Khunsa (intersex). These were rare and felt not applicable to most of the transgender community in Malaysia. In medically confirmed cases of Khunsa, the individual or parents of the individual would have to choose the most appropriate gender based on both physical and psychological characteristics; there was no acceptance of a third gender.

However, even amongst participants, there was a spectrum of opinion with an atypical minority of religious leaders discussing the need to be more flexible about issues such as transgenderism and specifically cited other Muslim countries, namely Iran which does permit gender reassignment surgery.

This is consistent with Islamic scholar Kamaili’s opinion, that although most religious leaders would conclude that there is no such thing as a third gender other than that of Khunsa there is the need to look at issues surrounding transgenderism and justice in Malaysia through the prism of compassion, fairness and science [24].

The spectrum of religious views shows that there is a window of debate that could be explored further. Malaysian scholars such as Ishak and colleagues have discussed medical treatment for transgender people outlining arguments of both supporters and opponents. [25]. The majority of religious leaders opposed gender reassignment surgery arguing on grounds based on ‘tampering with one’s God-made nature’ and changing one’s ‘social sexual role’ [25], consistent with some of the responses described in our study. The authors argue that proponents, mainly Shia as well as some Sunni jurists Shia deemed that sex change can be tolerated, and though unlawful it becomes permissible given the desperate need of transgender people under the remit of the legal maxim, “necessity overrides prohibition”.Also as a medical remedy, as it is not akin to changing one’s gender but more so “restoration of something amiss in him/her” and thus seen as “seeking medication which is lawful in Islam”.Despite these arguments they concede that such discussions within the context of Iran may be unhelpful in Sunni Malaysia [25], however, it does allow for debate and introduces the importance of local custom, a principle in Shariah called *urf* [26]. In the case of permission of gender reassignment surgery in Iran, Alipour also explains how the Islamic principle of *ijtihad* (independent reasoning) was used in jurisprudence [20]. It is noteworthy that a number of participants mentioned how transgender women were more socially accepted in Malaysian culture in yesteryear as opposed to now, whilst also some mentioning that Islam is in tune with local custom.

One of the key themes identified in the study was the conviction to varying degrees, that transgender women could be either ‘cured’, ‘corrected’ or that they could ‘come back’ to their original gender. Individuals from the transgender community are invited to attend the voluntary Muhayam camp, which JAKIM expresses is a spiritual retreat to increase their faith in Islam. The camps are a contentious issue with the transgender community viewing it as a failure to acknowledge their alternate gender was not a choice that could be changed back or forth, or a lifestyle, but was a core part of their identity. Most recently, the qualitative study conducted by international organisation Human Rights Watch echoed a similar statement with transgender women who saw the camps as an overt attempt to change transwomen into men [21]. Furthermore, the camps create distrust amongst the transgender community and religious leaders and it is noteworthy that one participant from the Ministry of Health said they supported JAKIM’s attempts at ‘correction’. Such actions create a cultural shift in attitudes to transgender women which can infiltrate various sectors of society.

The findings from this study support the growing body of research surrounding stigma and discrimination against transgender women in Malaysia at various sectors in society, including police, religious leaders and in health care settings. There are also allegations of victimisation and violence by the police, mentioned by participants in our study consistent with findings by international organisations such as Human Rights Watch [21]. The instances of discrimination by police offered to us in interviews is also consistent with Malaysian authors such as Lee, who describes the current practice akin to ‘policing sexual morality’ [13]. There ought to be an investigation into these allegations, sensitization of police to transgender issues to minimise such future occurrences as has been previously recommended by HRW [21]. Such actions would also serve to reassure the transgender community that their needs are being taken seriously and not dismissing their fears.

Such occurrences are cause for concern in themselves, but one could argue that when transgender women face the real threat of stigma, discrimination by police of both civil and religious, their focus on health and HIV prevention ultimately become less of a priority, hampering HIV prevention activities. The study by Teh supports this argument, conducted with transgender women in Malaysia found that for the majority of respondents HIV/AIDS was not “a primary concern for them”, compared to issues such as discrimination [11].

In 2014 a report was conducted by the UN country team looking into the policy and legal environment related to HIV services in Malaysia which found that the many socio-economic and legal issues affecting transpeople created an “unreceptive” environment that deterred transgender women from using health facilities [27]. Our findings from this study indicate that the present climate towards transgender women hinders their willingness to access services, with some participants mentioning first-hand negative experiences with health care workers, seen as untrustworthy, unprofessional and “bad-mouthed”. Research undertaken within the Malaysian context suggests that transgender persons may have reservations about accessing any form of health services [17] as they are hesitant about the reception they will receive from health care workers, doctors and nurses and whether they will be classified as ‘male’ or ‘female’ in any documentation. More recently, a study conducted with transgender women in Malaysia by Gibson and colleagues found that although the government health system was free “many reported avoiding it due to past discriminatory experiences”, while those who had utilised government clinics reported “denied care, mistreated, asked to change their appearance, or felt they had to present as male in order to receive proper care” [22]. A number of authors have concluded that there needs to be greater education of healthcare workers on transgender issues, [28] an increase in the capacity of ‘health and social service providers’ [29] to provide a sensitive service more responsive to their needs [30].

Given the prevailing climate in mainstream health services, transgender women are more comfortable and ‘safe’ approaching and being approached by non-governmental organisations with regards to HIV prevention services, which has those from the transgender and sex worker community within it. Such peer to peer schemes offer greater access to the transgender community and are trusted, this ought to be expanded and capitalised upon to provide HIV prevention services, however, adequate resources are needed for this. UNAIDS advises that investments should be made to build the capacity of local transgender led organisations and networks as “partners in the development, implementation and monitoring of HIV programmes for transgender communities” [29]. For this to come to fruition, there needs to be meaningful dialogue between transgender women and other key stakeholders, such as the Ministry of Health and religious leaders.

## Conclusion

The situation of transgender women in Malaysia and HIV prevention is a highly sensitive and challenging environment for all stakeholders, given the Islamic context and current legal system. Despite this apparent impasse, there are practically achievable areas that can be improved upon to optimise HIV prevention services and the environment for transgender women in Malaysia.

Recommendations:

- Investment in peer to peer services
- Greater involvement of transgender women in HIV policymaking and prevention strategy
- Sensitization of the police and healthcare providers to the needs of transgender women, highlighting the need for non-discrimination
- Full investigation of discrimination against transgender women and formal database of alleged incidents against transgender women
- Greater dialogue between religious leaders and transgender women.
- Independent scholastic review of Islamic juridprudence of transgender women in Malaysia seen through a prism of compassion, mercy and health.

## Abbreviations

JAKIM: Jabatan Kemajuan Islam Malaysia
PLHIV: People Living With HIV
TG: Transgender
